# Primary cerebral melanoma of the frontal lobe: a case report and review of the literature

**DOI:** 10.1097/RC9.0000000000000632

**Published:** 2026-06-30

**Authors:** Ayla Kouli, Ahmad Al-Bitar, Maher Saifo

**Affiliations:** a Faculty of Medicine, Damascus University, Damascus, Syrian Arab Republic; b Department of Oncology, Al-Bairuni University Hospital, Faculty of Medicine, Damascus University, Damascus, Syrian Arab Republic

**Keywords:** brain neoplasm, frontal lobe, intracranial tumor, malignant melanoma, primary cerebral melanoma

## Abstract

**Introduction:**

Primary cerebral melanoma is an exceedingly rare intracranial neoplasm, accounting for a minuscule fraction of all brain tumors and melanomas. Originating from melanocytes within the central nervous system, its diagnosis is one of exclusion, requiring thorough investigation to rule out metastatic disease from cutaneous, uveal, or mucosal primary sites.

**Case presentation:**

We present a 58-year-old male who presented with a recent onset of headaches and right lower limb weakness. Neuroimaging revealed a large, solitary, hemorrhagic mass measuring 5 × 5 cm in the left frontal lobe with significant surrounding edema and a mild midline shift. The patient underwent a craniotomy for gross total resection of the tumor. Histopathological and immunohistochemical analysis (positive for S100, HMB-45, and Melan-A) confirmed malignant melanoma. A comprehensive systemic workup, including dermatological and ophthalmological examinations, endoscopy, and a whole-body PET scan, revealed no evidence of a primary melanoma elsewhere, confirming the diagnosis of primary cerebral melanoma.

**Clinical discussion:**

Primary cerebral melanoma remains a significant diagnostic and therapeutic challenge due to its rarity and resemblance to other intracranial lesions. Gross total surgical resection is the cornerstone of treatment and is associated with improved survival outcomes. A definitive diagnosis necessitates a comprehensive systemic evaluation to exclude a metastatic origin.

**Conclusion:**

This case underscores the critical importance of considering primary melanoma in the differential diagnosis of solitary brain masses. Complete resection offers the best prognosis, and adjuvant radiotherapy may improve local control. Emerging targeted therapies and immunotherapies hold promise but require further study in primary CNS melanoma.

## Introduction

Primary cerebral melanomas are rare intracranial tumors, representing approximately 1% of all melanomas and 0.07% of all brain tumors^[^1^]^. They exhibit a male predominance^[^2^]^. Malignant melanomas originate from melanocytes and typically present in the leptomeninges^[^3^]^. Central nervous system (CNS) melanomas usually represent metastases and are rarely primary^[^4^]^. Thus, the diagnosis of primary cerebral melanoma involves the exclusion of metastatic disease from skin, ocular (uveal), and mucosal sites, as well as metastasis from lung and breast primaries. Clinical manifestations include increased intracranial pressure, neurological dysfunctions, and hemorrhage^[^2^]^. The treatment of choice for local intraparenchymal melanomas is complete resection followed by radiochemotherapy^[^5^]^. This case report has been prepared in accordance with the SCARE 2025 guidelines. In this report, we present a rare case of primary cerebral melanoma in the left frontal lobe of a 58-year-old male.

HIGHLIGHTS

- This case underscores the critical importance of considering primary melanoma in the differential diagnosis of solitary brain masses.
- Gross total surgical resection is the cornerstone of treatment and is associated with improved survival outcomes.

## Case presentation

A 58-year-old Arab male presented with a recent onset of headache lasting a few days, accompanied by right lower limb weakness. His past medical history was unremarkable for any neurological complaints, chronic illnesses, or prior surgeries. He had no known drug allergies, and his family history was negative for melanoma or other malignancies. He denied any alcohol use or smoking. Neurological examination revealed right-sided motor weakness (grade 4/5 in the lower limb, grade 4+/5 in the upper limb), with preserved consciousness, speech, and cranial nerve function. The differential diagnosis at this stage included primary brain tumor (e.g., glioma, primary CNS melanoma), solitary metastasis, hemorrhagic lesion, or infectious process.

**Figure 1.**
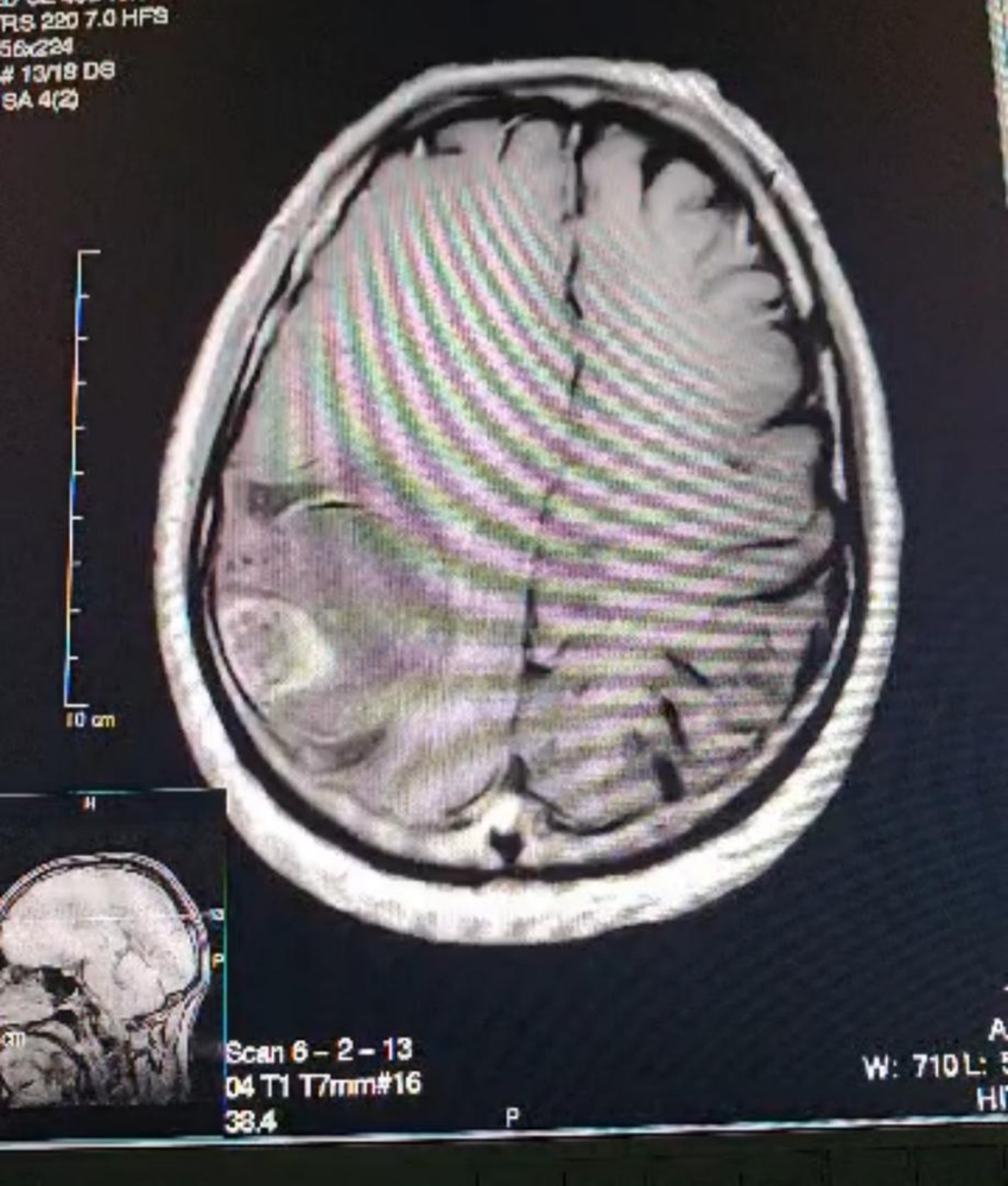
Computed tomography (CT) scan of the brain showing a well-circumscribed, hyperdense parenchymal lesion.

**Figure 2.**
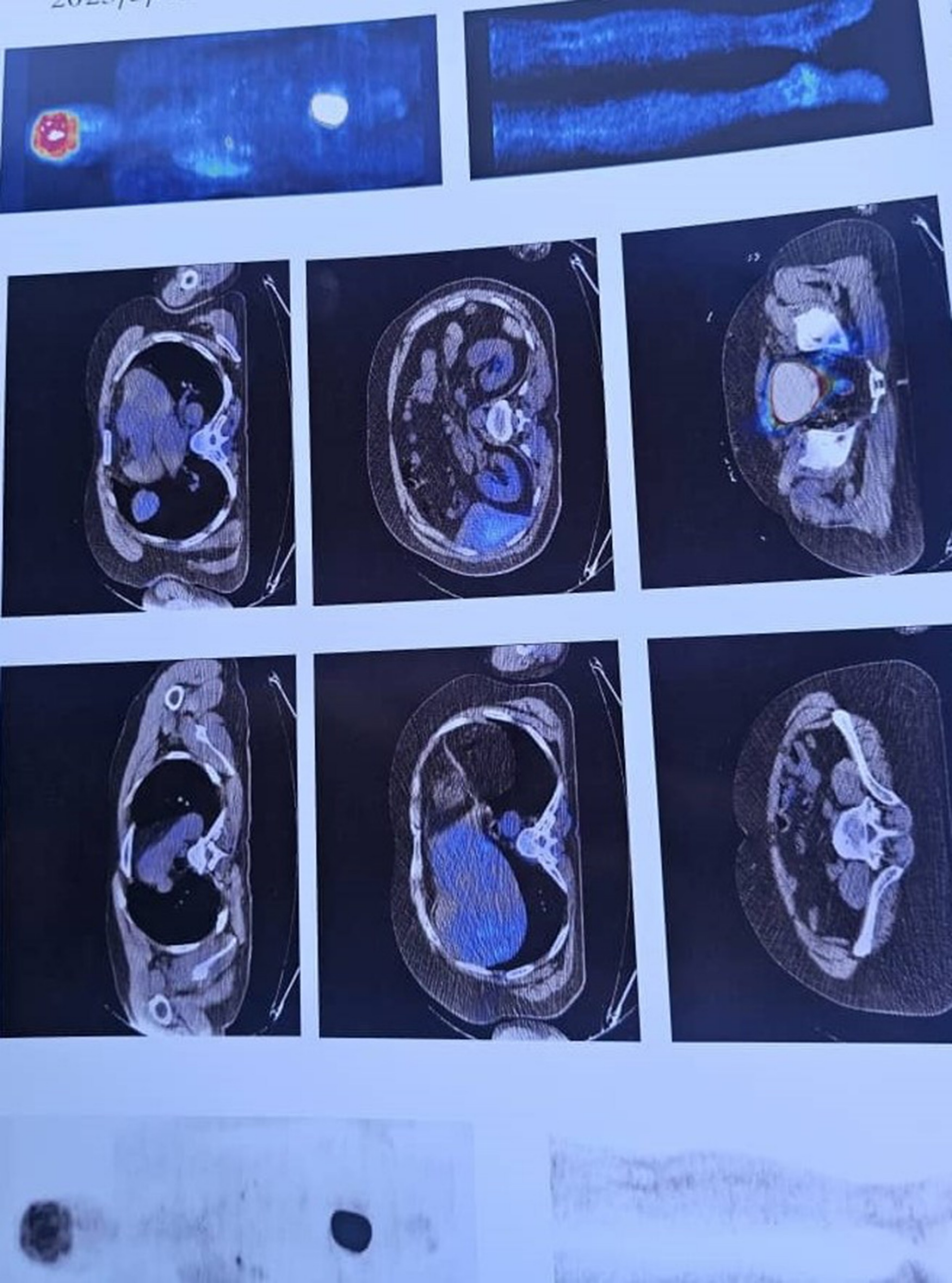
Magnetic resonance imaging (MRI) demonstrating avid homogeneous enhancement of the lesion, with associated perilesional edema.

Brain CT without contrast revealed a large, oval, heterogeneously marginated mass measuring approximately 5 × 5 cm, located within the white matter of the left frontal lobe, causing compression of adjacent frontal cortex structures and a slight midline shift (Figs 1 and 2). MRI with contrast confirmed a large left frontal lesion measuring 5 × 5 × 4.5 cm, displaying heterogeneous T1 and T2 signal intensities with surrounding edema and irregular peripheral enhancement (23 × 33 mm). The lesion exerted a mild pressure on the anterior horn of the left lateral ventricle but showed no evidence of herniation or leptomeningeal involvement. The posterior fossa and ventricles appeared normal.

The patient underwent a left frontal craniotomy. Intra-operatively, the mass was a hemorrhagic tumor with nodular areas; complete resection and hematoma evacuation were achieved with full hemostasis. Postoperatively, the patient was alert, ambulatory, and discharged in excellent neurological condition on antiepileptics and anticoagulants.

**Figure 3.**
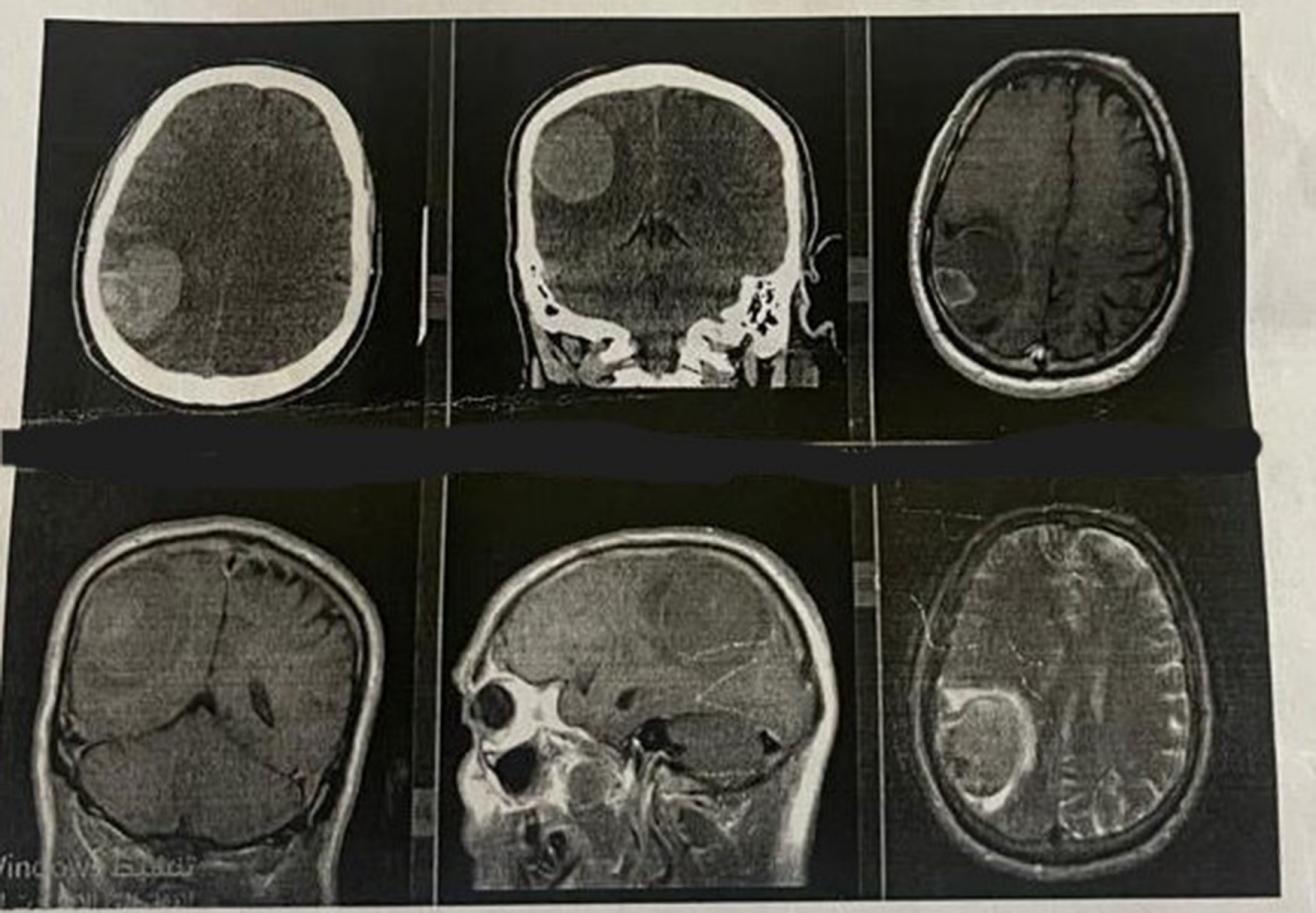
Whole-body positron emission tomography (PET) scan revealing no evidence of hypermetabolic activity outside the central nervous system, effectively ruling out systemic metastatic disease and supporting the diagnosis of primary central nervous system melanoma.

Histopathological examination revealed primary malignant melanoma. On H&E staining, the tumor was composed of sheets of epithelioid cells with abundant eosinophilic cytoplasm, moderate nuclear pleomorphism, prominent nucleoli, and occasional intracytoplasmic brown granular pigment consistent with focal melanin deposits. Scattered necrotic areas were observed. Mitotic figures were approximately 4 per 10 high-power fields. No vascular invasion was seen. Immunohistochemically, the tumor was positive for S100, HMB-45 (diffuse, strong cytoplasmic), and Melan-A (diffuse, strong cytoplasmic), with a Ki-67 proliferation index of approximately 15%. No molecular testing for BRAF mutations was performed due to a lack of tissue availability for additional analysis. Subsequent examinations were performed to rule out metastatic melanoma. These included a full dermatological physical examination, ophthalmological fundoscopic examination, endoscopy of the gastrointestinal tract, body CT, and a whole-body positron emission tomography (PET) scan. Findings revealed no evidence of melanoma in other parts of the body (Fig. 3). The patient was treated with postoperative radiotherapy and was advised to return immediately if seizures, fever, or signs of deep vein thrombosis occurred. Selected laboratory findings are shown in Table 1.

**Table 1:** Lab tests.

Test	Result	Normal Range	Status
Clotting time	14.9 s	≤13 s	↑ High
PTT (K.C.C.T)	52 s	26–36 s	↑ High
Blood glucose (fasting)	160 mg/dL	70–110 mg/dL	↑ High
WBC	14.9 × 10^3^/μL	5–10 × 10^3^/μL	↑ High
Neutrophils	90%	36–70%	↑ High
Lymphocytes	5%	24–44%	↓ Low
Hemoglobin	13.1 g/dL	13.5–16.2 g/dL	↓ Low
RBC	4.84 × 10^6^/μL	5–6 × 10^6^/μL	↓ Low
INR	1.29	-	Normal
Platelets	215 × 10^3^/μL	150–450 × 10^3^/μL	Normal

A postoperative MRI scan performed 6 weeks after surgery confirmed gross total resection with no evidence of residual tumor. The patient completed a standard course of adjuvant fractionated radiotherapy to the tumor bed (total dose: 60 Gy in 30 fractions). At the 12-month follow-up, the patient remained neurologically intact with no recurrence of symptoms. Surveillance MRI at 6 and 12 months postoperation showed stable postoperative changes without evidence of local recurrence or new intracranial lesions. The patient continues to be monitored with clinical and radiological evaluations every 6 months. Patient perspective (SCARE 2025 item 16) was not obtained because the patient was lost to follow-up after the 12-month visit and could not be reached for comment despite reasonable attempts.

## Discussion

Melanocytes are of neural crest origin and can be found in numerous parts of the body, including the leptomeninges. Primary melanocytic CNS tumors include melanocytoma, leptomeningeal melanocytosis and melanomatosis, and primary malignant melanoma^[^6^]^. Virchow first described primary melanocytic neoplasms of the CNS in 1859. The global incidence of intracranial melanomas shows a tendency to increase year by year^[^7^]^. Primary CNS melanoma has an incidence rate of 0.01 per 100 000 individuals per year^[^8^]^. It has a male predominance and is usually seen in middle-aged to elderly age groups^[^9^]^. The diagnosis of primary cerebral melanoma is often difficult preoperatively^[^7^]^. Symptoms may include meningism, subarachnoid hemorrhage, focal neurologic deficits, intracranial hypertension, hydrocephalus, and seizures^[^10^]^. CT findings are usually non-specific and may present as a superficially located hyperdense mass^[^11^]^. Prognosis varies based on tumor site and extent^[^12^]^; therefore, accurate diagnosis is crucial.

Regarding the laboratory findings in Table 1, several abnormalities warrant comment. The elevated white blood cell count (14.9 × 10^3^/μL) with neutrophilia (90%) and lymphopenia (5%) likely reflects a physiological stress response to the intracranial mass or early postoperative inflammation rather than infection. Prolonged clotting time (14.9 seconds) and PTT (52 seconds) with a normal INR (1.29) may indicate mild coagulopathy or preanalytical variation; however, no bleeding complications occurred perioperatively. The fasting hyperglycemia (160 mg/dL) was an incidental finding and prompted further evaluation for new-onset diabetes mellitus. Mild anemia (hemoglobin: 13.1 g/dL, RBC: 4.84 × 10^6^/μL) was clinically insignificant. These abnormalities did not alter the acute management but highlight the need for metabolic and hematological monitoring in such patients.

The treatment of choice for primary cerebral melanoma is complete resection followed by postoperative radiotherapy or chemotherapy^[^13^]^. Other treatment options include immunotherapy with interferon (INF) beta or alpha-2b^[^12,14^]^. Drugs targeting BRAF mutant proteins have been developed for human melanoma^[^15^]^. However, the role of specific chemotherapeutic agents and their effects on primary CNS melanoma need further evaluation. Median survival for metastatic melanoma is only 5–6 months, whereas patients with primary CNS melanoma can sometimes survive over 15 years^[^10^]^. Better outcomes are seen with total resection rather than partial resection. Postoperative radiochemotherapy is recommended^[^8^]^. Emerging targeted and immunotherapies – such as BRAF/MEK inhibitors (vemurafenib, dabrafenib, and trametinib) and immune checkpoint inhibitors (nivolumab, pembrolizumab, and ipilimumab) – have revolutionized the treatment of metastatic melanoma, but data specifically for primary CNS melanoma remain limited to case reports and small series. For instance, Fujimori *et al* reported a patient with primary CNS melanoma and leptomeningeal spread who received vemurafenib followed by nivolumab but died 5 months after initiation^[^16^]^. Prospective studies or registry-based analyses are urgently needed to define the role of these agents in primary CNS melanoma, particularly in BRAF-mutant tumors.

Our review of the literature, summarized in Table 2, highlights several consistent themes. The demographic trend of male predominance and presentation in middle to elderly age groups aligns with our case of a 58-year-old male, consistent with larger series^[^7,9,17^]^. Maximal safe surgical resection remains the cornerstone of treatment across nearly all reported cases. Favorable outcomes following total resection^[^10,11,18^]^ contrast with the poorer prognosis associated with partial resection or advanced disease^[^4,16,19^]^. This underscores the critical importance of achieving gross total resection, which was successfully accomplished in our patient. The role of adjuvant therapy remains less standardized. While radiotherapy is commonly employed postoperatively, as in our case and others^[^8,20^]^, the benefit of specific chemotherapy regimens is less clear^[^13,17^]^. The wide range of survival outcomes – from rapid progression within months^[^4,19^]^ to long-term survival exceeding 15 years^[^9,10^]^ – reflects the aggressive biological potential of some lesions and the critical impact of complete resection. Our patient’s excellent early postoperative outcome and 12-month disease-free status are consistent with the favorable trajectory associated with complete surgical excision and adjuvant radiotherapy.

**Table 2:** Literature review.

Authors (year)	Number of cases	Patient age	Gender	Tumor location	Treatment	Survival outcome
Rodriguez y Baena *et al* (1992)^[^18^]^	Review (1 case + lit review)	-	-	Intracranial solitary melanoma (mimicking meningioma)	Surgical case(s)	Favorable outcome with total resection^[^18^]^ (PubMed)
Greco Crasto *et al* (2001)^[^10^]^	1 + literature review	74 y	Male	Left Temporal lobe	Total Resection	2 years disease free^[^10^]^ (PubMed)
O’Connor *et al* (2007)	5 cases	Mean 35 y	3 women, 2 men	Solitary intraparenchymal lesions	Surgical treatment	-^[^21^]^ (PubMed)
Ma *et al* ^[2011,^^1^^]^	1 (single case)	15 y	Female	Intracranial	Not specified	Died of aggressive disease (PubMed)^[^1^]^
Gempt *et al* (2011)^[^4^]^	1	71 y	Female	Right frontal lobe.	Complete resection/ radiation	Progression/ Death after 18 months (PubMed)^[^4^]^
Fujimori *et al* (2012)	1 case	37 y	Male	Left Temporal lobe/leptomeninges	Partial resection + whole-brain RT + vemurafenib then nivolumab	Died 5 months after initiation^[^16^]^ (PubMed)
Somers *et al* (2013)^[^13^]^	1	65 y	Female	Arising from pineal gland	Neuroimaging diagnosis	Not specified (PubMed)^[^22^]^
Shah *et al* (2013)^[^11^]^	1	28 y	Female	Left middle cranial fossa	Total Resection	Symptom free 4 y postoperation^[^11^]^ (PubMed)
Wang *et al* (2014)^[^20^]^	Surgical Case series (8 patients)	-	-	Intracranial melanoma	Microsurgery and radiotherapy	The average follow-up duration was 13.8 months. Three patients died from this disease. One patient suffered from recurrence at the 16th month and underwent second surgery. The other patients were still alive with no evidence of tumor recurrence^[^20^]^ (PubMed)
Terao *et al*; literature review (1989–2014)	19 cases	Mostly middle-aged to elderly	Predominantly male	Cerebrum (various sites); 3 in cerebello-pontine angle	Excision; some with adjuvant chemo/radiotherapy	5 died < 1 year; one survived 17 years^[^9^]^ (PMC)
Suranagi *et al* (2015)^[^9^]^	1 + lit review	65 y	Male	Right parafalcine frontal region	Total Resection	Improved postoperation^[^9^]^ (PubMed)
Naik *et al* (2016)^[^23^]^	1	35 y	Female	Left parietal	Total Resection	-^[^23^]^ (PubMed)
Li *et al* (2019) – 115 cases pooled (15 own + 100 literature)	115 cases	Diffuse lesions younger (statistically)	Not specified	Diffuse vs solitary patterns	Surgery, radiotherapy, chemotherapy	Mean follow-up 16.6 months; 76 deaths (66.1%)^[^17^]^ (PubMed, Journal of Neurosurgery)
Lim *et al* (2019)	1 case	62 y	Male	Left temporal lobe	Gross total resection + radiotherapy (28 fr); then VP-shunt + temozolomide	Deterioration and death from progressive disease^[^19^]^ (PubMed)

The strength of this report lies in the comprehensive diagnostic workup performed to definitively rule out a metastatic origin, including thorough dermatological, ophthalmological, endoscopic, and whole-body PET imaging. This rigorous exclusion process is essential for confirming the diagnosis of primary cerebral melanoma. Furthermore, the detailed documentation of surgical and adjuvant management provides a clear treatment paradigm. However, this study has limitations inherent to a single-case report. The follow-up period of 12 months, while showing an excellent initial outcome, is relatively short given the potential for late recurrence. The generalizability of our findings is limited by the rarity and heterogeneity of this tumor. Lastly, while we performed a narrative review of the literature, a formal systematic review and meta-analysis were beyond the scope of this case report but are needed to draw more definitive conclusions about optimal management strategies.

## Conclusion

Primary CNS melanoma is an extremely uncommon tumor of the central nervous system. It has limited treatment options, and better survival outcomes are associated with complete tumor resection. Therefore, considering primary CNS melanoma in the differential diagnosis of brain tumors is crucial for accuracy and improved outcomes. It is essential to rule out metastatic melanoma to confirm the diagnosis. Emerging therapies, including chemotherapy, immunotherapy, and targeted agents (e.g., BRAF/MEK inhibitors and immune checkpoint inhibitors), may provide more effective treatments for malignant intracranial melanomas, although their role in primary CNS melanoma requires further investigation.

## Ethical approval

Not applicable.

## Consent

Not applicable.

## Data Availability

The data that support the findings of this study are available from the corresponding author upon reasonable request.
